# Effects of acute cannabis inhalation on reaction time, decision-making, and memory using a tablet-based application

**DOI:** 10.1186/s42238-024-00215-1

**Published:** 2024-02-03

**Authors:** Ashley Brooks-Russell, Julia Wrobel, Tim Brown, L. Cinnamon Bidwell, George Sam Wang, Benjamin Steinhart, Gregory Dooley, Michael J. Kosnett

**Affiliations:** 1 Injury and Violence Prevention Center, Colorado School of Public Health, University of Colorado Anschutz Medical Campus, 13001 E. 17Th Place, Aurora, CO 80045 USA; 2 Department of Biostatistics and Informatics, Colorado School of Public Health, University of Colorado Anschutz Medical Campus, Aurora, CO USA; 3 Driving Safety Research Institute, University of Iowa, Iowa City, IA USA; 4 Institute of Cognitive Science, University of Colorado, Boulder, CO USA; 5 Department of Pediatrics, CU School of Medicine, University of Colorado Anschutz Medical Campus, Aurora, CO USA; 6 Department of Environmental and Radiological Health Sciences, Colorado State University, Fort Collins, CO USA; 7 Department of Medicine, CU School of Medicine, University of Colorado Anschutz Medical Campus, Aurora, CO USA; 8 Department of Environmental and Occupational Health, Colorado School of Public Health, University of Colorado Anschutz Medical Campus, Aurora, CO USA; 9 Department of Biostatistics and Bioinformatics, Rollins School of Public Health, Emory University, Atlanta, GA USA

**Keywords:** Drug impairment, Cannabis use, Drug tolerance, Reaction time, Psychomotor performance cannabis-impaired driving

## Abstract

**Background:**

Acute cannabis use has been demonstrated to slow reaction time and affect decision-making and short-term memory. These effects may have utility in identifying impairment associated with recent use. However, these effects have not been widely investigated among individuals with a pattern of daily use, who may have acquired tolerance. The purpose of this study was to examine the impact of tolerance to cannabis on the acute effects as measured by reaction time, decision-making (gap acceptance), and short-term memory.

**Methods:**

Participants (ages 25–45) completed a tablet-based (iPad) test battery before and approximately 60 min after smoking cannabis flower. The change in performance from before to after cannabis use was compared across three groups of cannabis users: (1) occasional use (*n* = 23); (2) daily use (*n* = 31); or (3) no current use (*n* = 32). Participants in the occasional and daily use group self-administered *ad libitum*, by smoking or vaping, self-supplied cannabis flower with a high concentration of total THC (15–30%).

**Results:**

The occasional use group exhibited decrements in reaction time (slowed) and short-term memory (replicated fewer shapes) from before to after cannabis use, as compared to the no-use group. In the gap acceptance task, daily use participants took more time to complete the task post-smoking cannabis as compared to those with no use or occasional use; however, the level of accuracy did not significantly change.

**Conclusions:**

The findings are consistent with acquired tolerance to certain acute psychomotor effects with daily cannabis use. The finding from the gap acceptance task which showed a decline in speed but not accuracy may indicate a prioritization of accuracy over response time. Cognitive and psychomotor assessments may have utility for identifying impairment associated with recent cannabis use.

**Supplementary Information:**

The online version contains supplementary material available at 10.1186/s42238-024-00215-1.

Acute cannabis use has been demonstrated to affect psychomotor performance and cognitive functioning (McCartney et al. 2021). These impairing effects have public health relevance for traffic safety, occupational safety, and injury prevention (Hall et al. 2019). For example, systematic reviews have associated acute (recent) cannabis use with nearly a twofold increase in motor vehicle crash risk, including fatal crashes (Asbridge et al. 2012; Rogeberg 2019). Meanwhile, delta-9-tetrahydrocannabinol (THC, the primary psychoactive component of cannabis) is the second most frequently detected drug in fatally injured drivers in the USA (Li et al. 2011).

There is a critical need for objective assessments that identify impairment from cannabis. Blood THC level is poorly correlated with impairment and cannot always discriminate against an individual who has used cannabis recently or their degree of impairment, despite being widely included in impaired driving policies (Governors Highway Safety Association 2023; Arkell et al. 2021; Wurz and DeGregorio 2022). Part of the challenge of using blood THC to indicate impairment is due to the potential for acquiring tolerance to some of the impairing effects of THC (Broyd et al. 2016; Colizzi and Bhattacharyya 2018; Ramaekers et al. 2011; McCartney et al. 2022). A recent systematic review and meta-analysis concluded that the impairment effects of cannabis depend on the history of previous cannabis use, with a lesser magnitude and shorter duration of effects observed in those with a pattern of daily and frequent use as compared to those with a pattern of occasional use (Colizzi and Bhattacharyya 2018; McCartney et al. 2022).

Looking beyond biological samples like blood, there is growing attention to behavioral and cognitive assessments as an approach to detecting impairment. In the context of motor vehicle safety and roadside assessment, the most common approach to detecting impairment at the roadside has been the Standard Field Sobriety Test (National Highway Traffic Safety Administration 2023). It is widely used by law enforcement because of its relevance for alcohol impairment. However, it yields a relatively inaccurate assessment of impairment associated with cannabis use (Papafotiou et al. 2005; Downey et al. 2012; Spindle et al. 2021). Emerging research is examining portable computer tablet and cellphone-based applications testing cognitive and psychomotor performance associated with acute cannabis use (Chung et al. 2020; Karoly et al. 2022; Pal et al. 2016). If shown to be effective, such tools hold great promise for detecting impairment in settings ranging from workplaces to the roadsides. A standardized and mobile assessment could be deployed post-incident, such as after a motor vehicle crash, or to prevent such an incident, such as in a fitness for duty assessment.

There are some common approaches to the behavioral assessments of drug impairment. One measure that is commonly included in psychomotor and cognitive test batteries is a measure of reaction time. Reaction to visual stimuli is highly relevant for motor vehicle safety and collision avoidance. A recent meta-analysis found acute cannabis use was associated with slower reaction time (McCartney et al. 2021). The same meta-analysis found acute cannabis use may also adversely impact short-term or working memory. Working memory is increasingly understood to be important for safe driving. A systematic review of working memory and driving studies, inclusive of simulator and on-road studies, concluded that the greater the working memory capacity, the better the driving outcomes (Zhang et al. 2023). Additional known effects of acute cannabis use include changes to executive function, judgment or decision-making, fine motor control, and spatial judgment (McCartney et al. 2021), all of which have implications for motor vehicle safety and could be utilized as objective markers of impairment.

In light of the growing body of literature on tolerance to cannabis use (Colizzi and Bhattacharyya 2018; Ramaekers et al. 2011, 2009; Broyd et al. 2016; Desrosiers et al. 2015), it is critical to assess the extent to which tolerance to drug effects may impact the assessment of acute drug effects (Colizzi and Bhattacharyya 2018; Figueiredo et al. 2020). The purpose of this study was to investigate the utility of a tablet-based cognitive and psychomotor test battery to discern changes in performance associated with acute cannabis use among participants with a pattern of occasional and daily cannabis use. We used an observational design with participants who smoked self-supplied, cannabis flower product reflecting what is typically available in US states and Canada with a legal retail cannabis market.

## Methods

### Participants

We recruited healthy adults between the ages of 25 and 45 between October 2018 and February 2020. Key exclusion criteria included history of drug or alcohol dependence, body mass index above 35 kg/m^2^, color blindness, currently pregnant, and employment in a job with shift work or overnight shifts. Based on self-reported patterns of typical cannabis use, participants were enrolled into one of three groups: (A) daily cannabis use, defined as smoking or vaping cannabis flower product at least one time per day, every day of the week for 30 days prior to enrollment; (B) occasional cannabis use, defined as smoking or vaping cannabis flower product on at least 1 day but no more than 2 days per week in the 30 days prior to enrollment; and (C) nonusers, defined as at least one-lifetime cannabis use but no use in the month prior to enrollment. Additional details about recruitment and screening procedures have been previously published (Brooks-Russell et al. 2021).

### Procedures

The study used an observational design, comparing psychomotor and cognitive performance on the computer tablet-based Vitals assessment (Edmonton, Canada), before and after smoking cannabis flower, and compared results across three groups of cannabis use (occasional daily and no current cannabis use). Prior work has validated the Vitals with older adults and commercially licensed truck, bus, and light vehicle drivers (Bakhtiari et al. 2020; Scott et al. 2023). The study was observational in nature in that participants self-supplied the cannabis flower they used, and self-administered the cannabis, *ad libitum*. To account for learning effects, we included a comparison group of non-users that completed the same protocol, a baseline trial and then a second trial, without smoking cannabis. Participants were instructed not to use inhaled cannabis for at least 8 h and not to use edible cannabis for at least 12 h before the data collection appointment. Participants completed an alcohol breath test and provided a urine sample to verify abstinence from recent alcohol or drugs other than cannabis (30 mL Alere brand 13-panel iCup®). Participants then completed baseline assessments including Vitals.

Participants in the occasional and daily use groups were observed smoking or vaporizing cannabis flower while seated in a ventilated room. Participants smoked their own cannabis flower which was brought in its original packaging from a state-licensed Colorado dispensary to verify the percent total THC (required to be between 15 and 30%, and less than 2% cannabidiol (CBD) by weight) printed on the product label in accordance with state regulations. During a 15-min interval, participants smoked *ad libitum* with the instruction to smoke “the amount you most commonly use for the effect you most commonly desire.” Smoking occurred via a pipe, joint (rolled cigarette), bong, or vaporizer according to the participant’s choice. Participants in the no-use group were invited to relax for the equivalent amount of time.

Prior to use, and 30 min after the start of smoking (15 min after the end of the smoking period), a certified phlebotomist collected approximately 10 mL of whole blood using standard phlebotomy techniques to verify change in blood THC. The study was approved by the Colorado Multiple Institutional Review Board.

### Tablet-based measures

The Vitals assessment was conducted with an Apple iPad (Apple iPad 9.7″ 5th Gen Wi-Fi Only (Model A1822) Installed iOS version 11.4.1) mounted on a portable stand. The assessment consisted of different tasks, each with repeated trials, taking 10–18 min to complete, depending on how quickly participants moved through instructions and demonstrations. The tasks were designed to measure reaction time, gap acceptance (executive function), and working memory. The assessment was initiated approximately 58–89 min after the end of the smoking session (or equivalent time for non-users). This timing was due to other study assessments, which included performance in a driving simulator, and to approximate a real-world assessment of impairment, which would occur an hour or more after smoking and after an event leading to a crash or injury.

#### Task 1: Reaction time

**Fig. 1:**
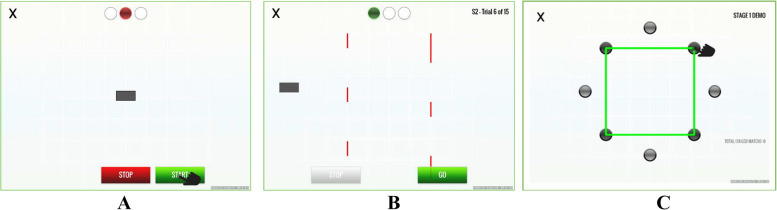
Screen from task demonstration developed by Impirica: **A** simple reaction time, **B** gap acceptance, and **C **memory

In the first task, *simple reaction time* is measured by presenting the participant with a bar in the middle of the screen and a “start” and “stop” button at the bottom. The participant presses the start button with a finger on their dominant hand, and after a period that randomly varied between 1.3 and 5.4 s, the bar begins to move horizontally across the screen, to the right or left. The goal is for the participant to move their finger from the start button to the stop button as quickly as possible after the bar begins to move (see Fig. 1). Reaction time is the time interval (in milliseconds) between the start of the bar movement and the participant touching the stop button. After the first 10 series of trials in which the bar only moves to the right, the task becomes a *choice reaction time* in which the bar can move in either direction, and the participant has to choose the stop button concordant with the direction of the bar, thus increasing the difficulty of the task. The task consisted of 25 completed trials. Trials in which the participant pressed the stop button prematurely, i.e., before movement of the bar, or in the incorrect direction, were not counted. Trials in which the subject omitted a response were counted as omission errors. The mean and standard deviation of the usable trials were calculated separately for the simple reaction time (Task 1a), choice reaction time (Task 1b), and all trials combined (Task 1c), with a precision of at least 60 fps (17 ms). Lower values represent faster reaction time.

#### Task 2: Gap acceptance

In the second task, the participant used “start” and “stop” buttons to move a small rectangular box horizontally across the screen as quickly as possible, without the bar hitting a series of lines of variable length that are moving vertically across the path of the box. The participant had to assess the size and timing of the varying gaps between the moving vertical lines and to move the box across the screen successfully through the gaps without hitting the lines. The instructions were as follows: “Use the buttons to move the box through the moving lines without touching them. While it is important to do this as quickly as possible, it is more important to get through safely.” For the first 10 trials, there was one set of lines moving vertically across the screen and in 10 subsequent trials there are two sets of lines to navigate (see Fig. 1). The participant could not start until presented with a green “go” button but could start and stop the bar while moving across the screen. The omission of a response within the 20 s allocated for a trial was counted as an omission error. Each participant completed 20 total trials (not including trials with premature starts). From this task, we measured the following: (Task 2a) the number of premature starts, divided by total trials, with lower numbers representing improved performance; (Task 2b) the total time in seconds to complete trials (not including premature starts) with lower values representing faster (improved) performance; and (Task 2c) number of successful trials in which the box did not strike the moving vertical lines divided by the total number of trials (including premature starts and trials with omission errors), with a higher proportion representing improved performance.

#### Task 3: Working memory

In the third task, the participant is briefly presented with a shape (triangle, rectangle, or polygon) that connects a series of dots. Next, a dark distractor screen appears for 2 s, and then the dots re-appear without the shape. The participant’s goal is to recreate the shape by tracing with their finger on the touchscreen within a 15-s interval (see Fig. 1). A single shape is shown in 6 trials, followed by 6 more difficult trials in which two shapes are successively shown. Trials in which there were no responses in the allocated time were tabulated as omission errors. In subjects with omission errors, the total time to complete the replication was normalized to 6 single shape trials and 6 two shape trials. Although the participant was only instructed to replicate the shapes as accurately as possible, we explore time to complete the task. Outcome measures include (Task 3a) the total number of correctly drawn shapes from all trials and (Task 3b) the total time to complete the shape replication, regardless of accuracy.

### Analysis

We derived quantitative outcomes from the three tasks for baseline and post-cannabis smoking. Outcomes were analyzed using linear regression models with the primary independent variable being the use group (no current use, occasional use, and daily use). Gender and age were included as potential confounders; age has been shown to be an important predictor in other tablet-based assessments (Bakhtiari et al. 2020) and there may be gender differences in reaction time (Blough and Slavin 1987; Adam 1999) and working memory (Hill et al. 2014). For the primary analysis, the dependent variable was the intra-individual change in the score of the outcome value post-cannabis minus the outcome value at baseline. The difference in the covariate-adjusted least-squared mean between the baseline period and the post-period was calculated and assessed for statistical significance for each user group. Baseline versus post-period least squared mean differences for each user group were contrasted with each other (occasional use versus no-use, daily use versus no-use, and occasional versus daily use) to assess the significance of cannabis use history on change in performance. We also assessed the impact of cannabis use history on performance by calculating the standardized mean difference of the baseline to post-use change in unadjusted outcome measures among user groups. The significance threshold was set at* p* < 0.05. All analyses were conducted using R version 4.3.1. (R Core Team. R 2013) and least squared mean differences and standardized effect sizes were produced using the emmeans package (Lenth 2018). The datasets used and/or analyzed during the current study are available from the corresponding author upon reasonable request.

## Results

### Participants

Eighty-six healthy adults (43 men, 43 women, ages 25 to 45; 31 with daily use, 23 with occasional use, and 32 with no current use) completed the study (Table 1). Participants in the daily use group reported cannabis use on 29.7 (SD = 1.3) of the past 30 days, and a mean of 5.0 (SD = 4.6) times a day. Participants in the occasional use group reported using a mean of 5.5 (SD = 2.5) days in the past 30 days, 1.5 (SD = 0.5) days in a typical week, and 1.4 (SD = 0.9) times per day on the days used (data not shown). The occasional and daily use group used products with similar concentrations of total THC. Specifically, the occasional use group used flower labeled with a range of 15.3 to 29.7% total THC, and an average concentration of 21.1%. The daily use group used flower labeled with a range of 15.0 to 27.5% total THC, with an average of 22.1% total THC. Blood samples taken before and after smoking confirmed that participants inhaled cannabis as requested; as expected the no-use group had no detectable THC or other cannabinoids in their blood at baseline. Among the daily use group, the mean baseline blood THC level was 5.0 ng/mL (range =  < LOD–26.0), which rose to 36.0 ng/mL (range = 1.3–146.7) at 30 min after the start of smoking. Among the group using cannabis occasionally, the mean baseline blood THC was non-detectable (< LOD = 0.2 ng/mL) which rose to 6.6 ng/mL (range = 1.0–29.6) at post-use (data not shown; previously published (Brooks-Russell et al. 2021)).

**Table 1:** Participant demographic characteristics and cannabis use experience

	**No current use (*N* = 32)**	**Occasional use (*N* = 23)**	**Daily use (*N* = 31)**	**Overall (*N* = 86)**
**Gender**				
Male	13 (40.6%)	13 (56.5%)	17 (54.8%)	43 (50.0%)
Female	19 (59.4%)	10 (43.5%)	14 (45.2%)	43 (50.0%)
**Age**				
25–35 years	20 (62.5%)	19 (82.6%)	21 (67.7%)	60 (69.8%)
36–45 years	12 (37.5%)	4 (17.4%)	10 (32.3%)	26 (30.2%)
**Number of days used, past 30**				
Mean (SD)	--	5.52 (2.45)	29.7 (1.27)	12.3 (13.4)
Median [Min, Max]	--	5.00 [2.00, 10.0]	30.0 [25.0, 30.0]	5.00 [0, 30.0]
**Times used per day on average, past 30**				
Mean (SD)	--	1.35 (0.94)	5.03 (4.63)	3.43 (3.96)
Median [Min, Max]	--	1.00 [1.00, 5.00]	4.00 [1.00, 25.0]	2.00 [1.00, 25.0]

### Group differences in performance from pre- to post-cannabis use

**Fig. 2:**
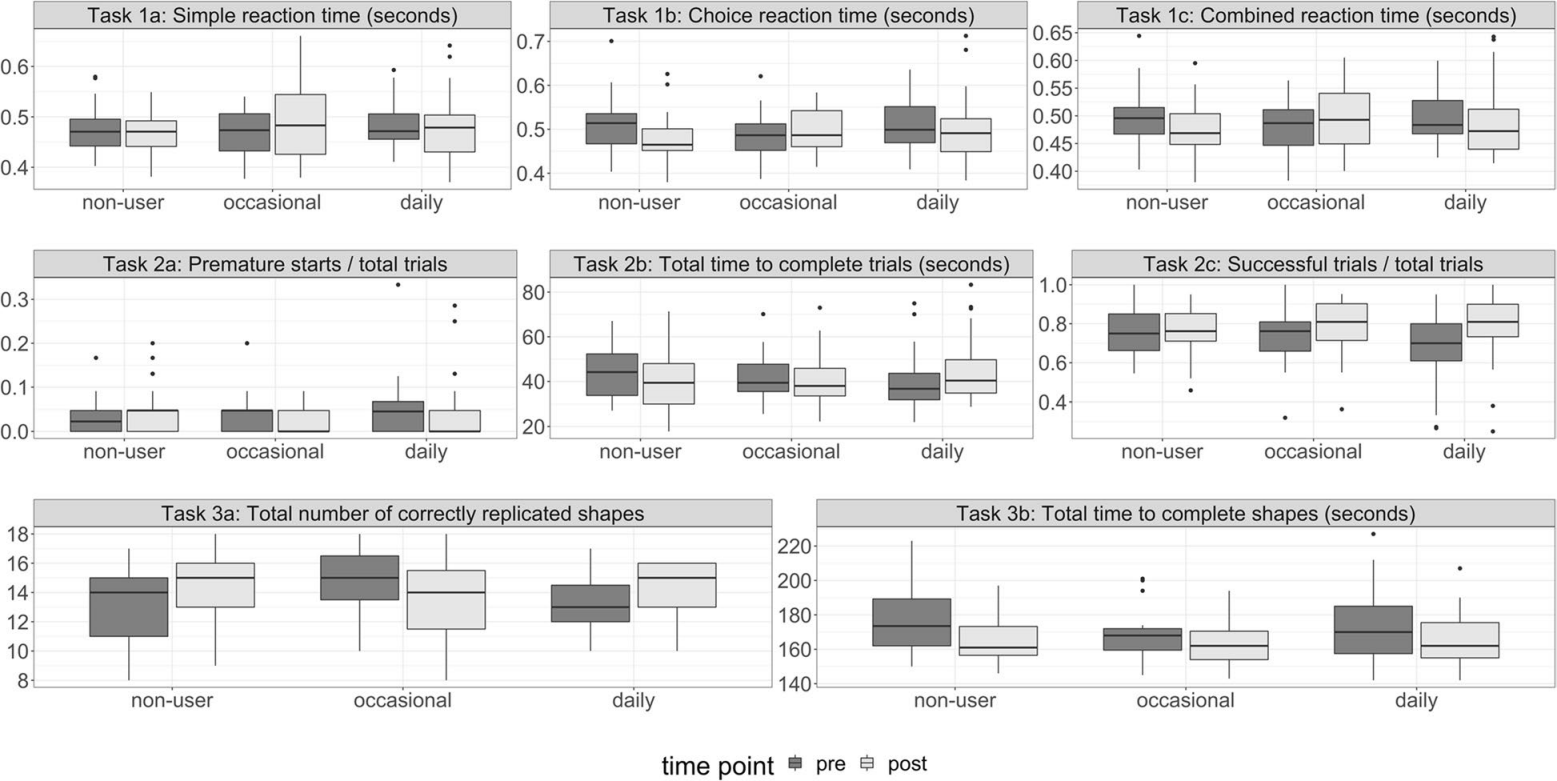
Boxplots of unadjusted mean outcome values by use group at pre- and post-time points

Table 2 presents the adjusted means of post-cannabis use performance minus pre-cannabis use performance for outcome variables across the three tasks. Gender and age were not influential control variables and thus the unadjusted values at pre and post, presented in Fig. 2 and Supplemental Table 1, are similar to the adjusted results in Table 2. Supplemental Table 2 presents a test of baseline difference. The total number of replicated shapes significantly differed at baseline, with the occasional use group replicating more than the non-use or daily use group (*p* = 0.02). There was a marginally significant difference (*p* = 0.05) at baseline between the daily use and non-use group in the success ratio variable. Otherwise, values tend to be relatively similar across groups at baseline. The pattern of findings for the simple and choice reaction time trials were consistent so we focus on the results that combined all trials due to the increased power from the larger number of trials (Task 1c). Adjusted performance improved in reaction time trials (Task 1c), from pre- to post-assessment among those with no cannabis use, as evidenced by the negative value of − 20.76 ms (post–pre; 95% CI =  − 34.22, − 7.3), whereas there was a worsening of performance among the occasional use group of nearly the same magnitude (mean = 17.15 ms; 95% CI = 1.11, 33.18) and less change among the daily use group (mean =  − 6.75 ms; 95% CI =  − 20.44, 6.94). The difference between the occasional use group and no-use group was statistically significant (*p* < 0.001), as was the difference between the occasional and daily use group (*p* = 0.03).

**Table 2:** Change in post-use minus pre-use (baseline) performance assessment by user group, adjusted for age and gender

	No current use (post-pre) Mean (95% CI)	Occasional use (post-pre) Mean (95% CI)	Daily use (post-pre) Mean (95% CI)	Overall *p* (ANOVA)	No-use vs. occ *p*	No-use vs. daily *p*	Occ. vs. daily *p*
**Task 1: Reaction time**							
1a: Simple reaction time (msec)	-9.86 (-29.85, 10.12)	27.68 (3.86, 51.49)	-3.28 (-23.6, 17.05)	**0.03**	**0.02**	0.65	0.05
1b: Choice reaction time (msec)	-28.15 (-46.79,-9.5)	9.41 (-12.8, 31.62)	-10.49 (-29.45, 8.47)	**0.03**	**0.01**	0.19	0.18
1c: Combined reaction time (msec)	-20.76 (-34.22,-7.3)	17.15 (1.11, 33.18)	-6.75 (-20.44, 6.94)	**.001**	** < .001**	.15	**.03**
**Task 2: Gap acceptance**							
2a: Premature starts ratio (number of premature starts divided by total trials)	0.02 (-0.01, 0.04)	-0.02 (-0.05, 0.01)	0 (-0.03, 0.02)	.10	.05	.34	.27
2b: Total time to complete trials (seconds)	-3.67 (-7.04,-0.29)	-1.30 (-5.18, 2.58)	4.61 (1.24, 7.98)	**.001**	.37	** < .001**	**.03**
2c: Success ratio (number of successful trials divided by total trials)	0.01 (-0.04, 0.06)	0.05 (-0.01, 0.11)	0.1 (0.05, 0.15)	.05	.29	**.01**	**.**19
**Task 3: Memory**							
3a: Total number of correctly replicated shapes	0.97 (0, 1.95)	-1.39 (-2.51,-0.27)	0.66 (-0.31, 1.63)	**.004**	**.002**	.65	**.008**
3b: Total time to complete shapes from memory (seconds)	-10.6 (-15.44,-5.76)	-4.38 (-9.93, 1.18)	-6.72 (-11.56,-1.89)	.20	.19	.30	.50

*msec* milliseconds, *occ* occasional

Post-minus pre-performance assesses change after acute use of cannabis for occasional and daily users. For non-users, it represents the change in performance in two consecutive sessions separated by an interval of relaxation. Bolded comparisons were statistically significant (*p* < 0.05)

In the second task (gap acceptance), in which participants made decisions about when to move a bar horizontally across the screen to avoid collisions with moving vertical lines with variable gaps, there was not a significant difference overall between groups in the number of premature starts (*p* = 0.10). There was a significant group difference for the total time to complete the trials (*p* = 0.001). Specifically, the no-use and occasional-use groups decreased the time needed to complete all the trials, as evidenced by negative post-use minus baseline values. By comparison, those with daily use required more time to complete the task after acute use of cannabis, as evidenced by a positive value (mean change post–pre: no-use =  − 3.67 s, 95% CI =  − 7.04, − 0.29; occasional use =  − 1.30 s, 95% CI =  − 5.18, 2.58; and daily use = 4.61 s, 95% CI = 1.24, 7.98). The difference between groups was significant for the contrast of no-use to daily use groups (*p* < 0.001) and between occasional and daily use groups (*p* = 0.03). The overall trial success ratio was relatively high for all groups in both the pre- and post-cannabis use intervals. At baseline, it was 68% for those with a pattern of daily use, 74% for those with occasional use, and 75% for those who do not currently use cannabis. At the post-assessment, it was 77% for those with no-use and 78% for those with occasional and daily use (Supplemental Table 1). There was a marginal (*p* = 0.05) significant overall group difference for the success ratio score (Table 2: Task 2c). This was driven by the significant contrast (*p* = 0.01) between the daily-use group compared to no-use; those in the daily-use group significantly improved performance after cannabis use, whereas those with no-use exhibited little change.

In the third task measuring working memory by recalling shapes, there was a significant overall group difference in the total number of replicated shapes (*p* = 0.004). Participants in the no-use and daily-use groups replicated more shapes at the post-test assessment (0.97 shapes, 95% CI = 0, 1.95; and 0.66 shapes, 95% CI =  − 0.31, 1.63; respectively) whereas the occasional use participants replicated fewer shapes (− 1.39, 95% CI =  − 2.51, − 0.27). The post-use decline in memory performance exhibited by the occasional use group was statistically significant in comparison to non-users (*p* = 0.002) and the daily use group (*p* = 0.008). There were no significant group differences in the total time to complete the memory task (*p* = 0.20).

## Discussion

This study examined the acute effects of smoked cannabis on psychomotor and cognitive performance, with a focus on the contrast between those who use cannabis occasionally and those who use it daily. In a systematic review, Colizzi and Bhattacharyyai concluded that cognitive and psychomotor effects of acute cannabis use are less pronounced for frequent users (Colizzi and Bhattacharyya 2018). Although frequent or regular use has not been consistently defined in the literature, daily use (often multiple times per day) represents a dosing pattern that appears most effective at eliciting downregulation and related changes in cannabinoid receptors that underly pharmacodynamic tolerance (Hirvonen et al. 2012). We hypothesized that following acute cannabis smoking, individuals who use cannabis daily, compared to occasionally, would exhibit less of an effect on their reaction time and less decrement in performance on the gap acceptance and working memory tasks. Consistent with this hypothesis of tolerance, we found that those in the occasional use group had a slower (longer) reaction time after acute cannabis smoking, which was a significant difference compared to the no-use comparison group, which had a shorter (faster) reaction time from baseline to the post-assessment. The daily use group did not exhibit an improvement in reaction time, and the differences between the daily and occasional or no-use groups were not statistically significant.

Reaction time tasks, in which an individual detects a start signal and responds, such as by pressing a button, evaluate a combination of information processing speed and movement execution. Long utilized as a psychomotor assessment tool, reaction time tasks have practical value because of the relative comparability of test design across platforms, and their relevance for safety-sensitive tasks such as driving. A recent meta-analysis by McCartney et al. (2021) examined the effect of acute cannabis use on reaction time in cognitive performance tests and driving outcomes. In an analysis that combined studies of participants with different cannabis use histories and different methods of use (oral, smoked, vaporized, and intravenous) they calculated small to medium adverse effects on reaction time assessed in neuropsychological testing and in studies of simulated and on-road driving performance (McCartney et al. 2021). However, this meta-analysis could not directly assess the effect of tolerance. In the few studies able to assess reaction time differences by the participant’s cannabis use history, the findings have been mixed. In two studies by Ramaekers and colleagues, they observed no significant impact of acute cannabis smoking on simple reaction in occasional users compared to near-daily/daily users (Ramaekers et al. 2009; Wel et al. 2013). However, Hunault et al. (2009) observed a dose-dependent increase (decline in performance) in simple reaction time in participants with occasional use tested at several time points after acute smoking of high-potency cannabis (Hunault et al. 2009).

Our finding that those with a history of occasional use replicated significantly fewer shapes on the working memory task as compared to those with daily or no-use is consistent with the hypothesis of acquired tolerance to cannabis impairment. Although an adverse impact of acute and chronic cannabis use on various aspects of memory has been extensively documented (Zhornitsky et al. 2021; Ranganathan and D’Souza 2006; Schoeler and Bhattacharyya 2013), few studies have directly compared the impact of acute cannabis smoking on working memory in participants with occasional vs. daily use. A placebo-controlled study of acute cannabis smoking on paired-associate learning found that participants who are daily/near-daily users exhibited no decline in performance after smoking two low-potency cannabis joints, whereas the same amount impaired the memory performance of people who smoked cannabis occasionally (Cohen and Rickles 1974; Rickles et al. 1973). In more recent research, smoking cannabis was associated with a decrement in accuracy on a high-load spatial working memory task in participants with a history of occasional use but not those with near-daily use (Hart et al. 2010; Ilan et al. 2004). Notably, despite not affecting the accuracy of spatial working memory in those with near-daily use, cannabis use increased response time in both groups (Hart et al. 2010; Ilan et al. 2004). After acute administration of 2.5 mg or 5.0 mg of intravenous THC, performance on a word recall task (D’Souza et al. 2008) and spatial working memory task (D’Souza et al. 2008) declined to a greater extent in those who were non-users than those with a history of frequent use. Desrosiers et al. observed no effect of acute cannabis smoking on the accuracy of spatial working memory in either participants with a history of occasional or near-daily/daily use; however, they were tested starting nearly 2 h after smoking, which may have limited the ability to detect significant effects (Desrosiers et al. 2015). In summary, our findings are consistent with the literature that suggests acquired tolerance to the effects of THC on working memory may occur with daily use.

The gap acceptance task contained in the Vitals battery assesses elements of executive function and processing speed. In this task, the daily use group exhibited a slowing of performance after acute cannabis use that was not observed in either the no-use or the occasional use group. Participants were instructed to prioritize accuracy over speed. Although the daily use group performed the task slower after acute cannabis use, on average their success ratio improved. The finding that individuals with daily cannabis use may retain accuracy at the expense of slowing the speed of performance has been noted in other studies (Hart et al. 2010; Greenwald and Stitzer 2000; Vadhan et al. 2007). This interpretation would be consistent with prior driving simulator studies in which driving slower is interpreted as compensatory cautiousness and reduced risk-taking (Brooks-Russell et al. 2021; Ramaekers et al. 2020). There was a notable lack of a significant effect of acute cannabis smoking on performance in the gap acceptance task by those with a history of occasional use. Inconsistent impacts of acute cannabis smoking on other measures of executive function (e.g., the Tower of London test) have been encountered (Ramaekers et al. 2009, 2006).

To our knowledge, this is the first study to compare the acute effects of cannabis on psychomotor performance in participants with a history of occasional and daily use after *ad libitum* smoking of cannabis typical of what is available in legal retail markets. In Colorado, the setting for the study, the average THC content in the retail market is 19% THC for flower and 80% THC for liquid concentrates (MPG Consulting 2019). Much of the extant literature has been conducted with lower THC concentration product, estimated to be an average of 6% THC (Burt et al. 2021), and a limited number of studies have used concentrations in the range of 10–13% THC. An *ad libitum* study design that allows for participants to self-titrate as they would normally smoke increases external generalizability and is particularly relevant for studies attempting to examine driving-related impairment.

This study has several limitations. First, the inability to randomly assign participants to use groups (e.g., daily, occasional, no current use) limits causal inference associated with the design. The use of an observational design in which participants supplied their own cannabis rather than an experimental design in which the investigator supplied the cannabis may have limited our ability to precisely quantify and control how much cannabis was smoked. Although this is an important limitation, this approach allowed us to study cannabis as typically consumed by participants using products available from licensed dispensaries. Even under experimental conditions with participants smoking supplied joints or inhaling cannabis via a vaporizer, there is evidence that research participants self-titrate consumption which undermines efforts to standardize the THC dose (Cooper and Haney 2009; Hartman et al. 2015). The tablet-based assessment was administered an hour or more after the end of the smoking period, which may have been after the most pronounced effects of smoked cannabis occurred. However, this timing is realistic for when an assessment like this would be given in a workplace or roadside setting, because of a delay from the use of cannabis to the accident or other event, and a time delay to when the individual would be assessed for impairment (Wood et al. 2016). Finally, participants may have been trying to perform as well as possible given they were being observed, which may lead to reduced differences in observed performance from pre to post.

## Conclusion

In two of the tablet-based tasks, the occasional use group performed slower (reaction time task) or less accurately (memory task) from before to after smoking, as compared to daily use and no-use groups, consistent with daily cannabis use resulting in tolerance to the acute effects of cannabis. In the gap acceptance task, the daily use group took longer to complete the task after smoking cannabis, while at the same time increasing their success ratio. Taken together, the findings are consistent with acquired tolerance to certain acute drug effects. The increase in time that the daily use group required to complete the gap acceptance task may nonetheless indicate an acute effect of cannabis among those in the daily use group, who prioritized accuracy over response time. Psychomotor and cognitive assessment batteries, such as the one used in this study, hold promise for providing an objective measure of cannabis impairment.

## Supplementary Information

**Additional file 1:** **Supplemental Table 1.** Unadjusted means of baseline and post assessment by use group. **Supplemental Table 2.** Test of baseline group differences, adjusted for age and gender.

## Data Availability

The datasets used and/or analyzed during the current study are available from the corresponding author on reasonable request.
