# Supplementary material for: Effects of acute cannabis inhalation on reaction time, decision-making, and memory using a tablet-based application
Source: J Cannabis Res. 2024 Feb 3;6:3. doi: 10.1186/s42238-024-00215-1 (PMC10837858; doi:10.1186/s42238-024-00215-1)
Supplement: Supplementary file 1 — Additional file 1: Supplemental Table 1. Unadjusted means of baseline and post assessment by use group. Supplemental Table 2. Test of baseline group differences, adjusted for age and gender. [file 42238_2024_215_MOESM1_ESM.docx]

**Supplemental Table 1**. Unadjusted means of baseline and post assessment by use group

|  | No current use  (PRE)  Mean (95% CI) | Occasional use  (PRE)  Mean (95% CI) | Daily use  (PRE)  Mean (95% CI) | No current use  (POST)  Mean (95% CI) | Occasional use  (POST)  Mean (95% CI) | Daily use  (POST)  Mean (95% CI) |
| --- | --- | --- | --- | --- | --- | --- |
| **Task 1: Reaction Time** |  |  |  |  |  |  |
| 1a. Simple reaction time (seconds) | 0.48 (0.46, 0.49) | 0.46 (0.44, 0.49) | 0.48 (0.47, 0.5) | 0.47 (0.44, 0.49) | 0.49 (0.47, 0.52) | 0.48 (0.45, 0.5) |
| 1b. Choice reaction time (seconds) | 0.51 (0.49, 0.53) | 0.49 (0.46, 0.51) | 0.51 (0.49, 0.53) | 0.48 (0.46, 0.5) | 0.5 (0.47, 0.52) | 0.5 (0.48, 0.52) |
| **Task 2: Decision-Making** |  |  |  |  |  |  |
| 2a: Number of premature starts as a fraction of total number of trials presented | 0.03 (0.01, 0.05) | 0.05 (0.02, 0.07) | 0.04 (0.02, 0.06) | 0.04 (0.02, 0.06) | 0.02 (0, 0.05) | 0.04 (0.02, 0.06) |
| 2b: Total time to complete trials (seconds) | 44.03 (40.09, 47.97) | 42.23 (37.58, 46.88) | 39.59 (35.58, 43.6) | 39.6 (35.01, 44.19) | 40.74 (35.33, 46.16) | 44.47 (39.8, 49.13) |
| 2c: Success ratio (number of successful trials divided by total trials) | 0.75 (0.7, 0.8) | 0.74 (0.67, 0.8) | 0.68 (0.63, 0.74) | 0.77 (0.72, 0.82) | 0.78 (0.72, 0.84) | 0.78 (0.73, 0.83) |
| **Task 3: Memory** |  |  |  |  |  |  |
| 3a: Total number of correctly replicated shapes | 13.19 (12.35, 14.02) | 14.91 (13.93, 15.9) | 13.39 (12.54, 14.23) | 14.03 (13.18, 14.89) | 13.52 (12.51, 14.53) | 14.03 (13.16, 14.9) |
| 3b: Total time to complete shapes from memory (seconds) | 175.47 (169, 181.94) | 167.91 (160.28, 175.55) | 172.71 (166.14, 179.28) | 165.09 (160, 170.19) | 163.61 (157.6, 169.62) | 165.74 (160.57, 170.92) |

**Supplemental Table 2**. Test of baseline group differences, adjusted for age and gender

|  | Non-use | Occasional | Daily | Overall P | P, non v. occ | P, non v. daily | P, occ v. daily |
| --- | --- | --- | --- | --- | --- | --- | --- |
| **Task 1: Reaction Time** |  |  |  |  |  |  |  |
| 1a. Simple reaction time (seconds) | 473.93  (456.35, 491.51) | 469.0  (448.8, 489.2) | 481.17  (463.59, 498.74) | 0.4 | 0.7 | 0.6 | 0.4 |
| 1b. Choice reaction time (seconds) | 507.38  (486.95, 527.8) | 490.15  (466.68, 513.61) | 512.48  (492.07, 532.89) | 0.2 | 0.3 | 0.7 | 0.2 |
| **Task 2: Decision-Making** |  |  |  |  |  |  |  |
| 2a: Number of premature starts as a fraction of total number of trials presented | 0.03  (0.01, 0.05) | 0.05  (0.03, 0.07) | 0.04  (0.02, 0.06) | 0.5 | 0.2 | 0.4 | 0.6 |
| 2b: Total time to complete trials (seconds) | 43.04  (39.11, 46.97) | 42.72  (38.2, 47.23) | 39.89  (35.96, 43.82) | 0.4 | 0.9 | 0.3 | 0.4 |
| 2c: Success ratio (number of successful trials divided by total trials) | 0.76  (0.71, 0.82) | 0.73  (0.67, 0.79) | 0.68  (0.63, 0.74) | 0.1 | 0.5 | 0.05 | 0.27 |
| **Task 3: Memory** |  |  |  |  |  |  |  |
| 3a: Total number of correctly replicated shapes | 13.12  (12.25, 13.99) | 14.77  (13.77, 15.77) | 13.49  (12.62, 14.36) | 0.02 | 0.02 | 0.6 | 0.06 |
| 3b: Total time to complete shapes from memory (seconds) | 174.62  (167.91, 181.32) | 169.26  (161.55, 176.97) | 171.58  (164.88, 178.29) | 0.4 | 0.3 | 0.5 | 0.7 |
